# Blue-light induced accumulation of reactive oxygen species is a consequence of the *Drosophila* cryptochrome photocycle

**DOI:** 10.1371/journal.pone.0171836

**Published:** 2017-03-15

**Authors:** Louis-David Arthaut, Nathalie Jourdan, Ali Mteyrek, Maria Procopio, Mohamed El-Esawi, Alain d’Harlingue, Pierre-Etienne Bouchet, Jacques Witczak, Thorsten Ritz, André Klarsfeld, Serge Birman, Robert J. Usselman, Ute Hoecker, Carlos F. Martino, Margaret Ahmad

**Affiliations:** 1 UMR CNRS 8256 (B2A), IBPS, Université Paris VI, Paris, France; 2 Department of Biomedical Engineering, Florida Institute of Technology, Melbourne, Florida, United States of America; 3 GCRN team, Brain Plasticity Unit, UMR 8249 CNRS/ESPCI Paris, PSL Research University, Paris, France; 4 Department of Physics and Astronomy, University of California, Irvine, California, United States of America; 5 Botany Department, Faculty of Science, Tanta University, Tanta, Egypt; 6 Department of Chemistry and Biochemistry, Montana State University, Bozeman, Montana, United States of America; 7 Botanical Institute and Cluster of Excellence on Plant Sciences (CEPLAS), Biocenter, University of Cologne, Cologne, Germany; 8 Department of Biology, Xavier University, Cincinnati, Ohio, United States of America; Syddansk Universitet, DENMARK

## Abstract

Cryptochromes are evolutionarily conserved blue-light absorbing flavoproteins which participate in many important cellular processes including in entrainment of the circadian clock in plants, *Drosophila* and humans. *Drosophila melanogaster* cryptochrome (DmCry) absorbs light through a flavin (FAD) cofactor that undergoes photoreduction to the anionic radical (FAD^•-^) redox state both *in vitro* and *in vivo*. However, recent efforts to link this photoconversion to the initiation of a biological response have remained controversial. Here, we show by kinetic modeling of the DmCry photocycle that the fluence dependence, quantum yield, and half-life of flavin redox state interconversion are consistent with the anionic radical (FAD^•-^) as the signaling state *in vivo*. We show by fluorescence detection techniques that illumination of purified DmCry results in enzymatic conversion of molecular oxygen (O_2_) to reactive oxygen species (ROS). We extend these observations in living cells to demonstrate transient formation of superoxide (O_2_^•-^), and accumulation of hydrogen peroxide (H_2_O_2_) in the nucleus of insect cell cultures upon DmCry illumination. These results define the kinetic parameters of the *Drosophila* cryptochrome photocycle and support light-driven electron transfer to the flavin in DmCry signaling. They furthermore raise the intriguing possibility that light-dependent formation of ROS as a byproduct of the cryptochrome photocycle may contribute to its signaling role.

## Introduction

Cryptochromes are a family of blue-UV/A light absorbing flavoprotein receptors found throughout the biological kingdom [1–3]. They are implicated in the regulation of growth and development in plants, and in the entrainment of the circadian clock in animals [4, 5]. A well-established biological function of *Drosophila* cryptochrome (DmCry) is to contribute towards setting the circadian clock in response to light [6]. It does so by binding to the *Drosophila* core clock protein Timeless (Tim) and the E3 ubiquitin ligase Jetlag in the presence of blue light. As a result, Tim is degraded and can no longer participate in its natural feedback loop involving interaction with the transcriptional activator complex Clock:Cycle (Clk:Cyc) [7]. In this way, the 24-hour internal oscillation is disturbed, and may even be completely stopped in constant light. DmCry has more recently been shown to have other functions independent of its role in the circadian clock, including direct light sensing in neurons [8] and response to stress [9].

The current paradigm for DmCry signaling is that DmCry undergoes a light- dependent conformational change which exposes binding sites for partner proteins such as Tim and Jetlag to initiate the signaling reaction. Crystal structure of full-length DmCry shows that a small (30aa) C-terminal extension is folded against an N-terminal light sensing domain, in the pocket close to the flavin cofactor [10, 11]. When this C-terminal extension is deleted, the partner proteins bind constitutively to the N-terminal domain of DmCry and therefore the response becomes constitutive. These observations indicate that the C-terminal domain of DmCry is released from the protein surface upon illumination, allowing partner proteins access to the N-terminal domain in a light-dependent manner [6, 7]. A similar paradigm involving light-initiated conformational change appears to hold for cryptochrome responses in other systems [1 – 5].

The current challenge in the field is to define the nature of the photochemical reaction which gives rise to the conformational change initiating the signaling response. Like all cryptochromes, DmCry binds a light-sensing FAD cofactor within a hydrophobic pocket adjacent to the C-terminal domain of the protein [10,11]. The flavin redox state is in the oxidized form in the purified protein when maintained in the dark. Illumination results in photoreduction to the anionic FAD^•-^ redox state [12 – 14]. These redox state transitions have been shown to accompany a light-induced conformational change and initiate biological signaling both *in vitro* [15] and *in vivo* [16], Therefore, the anionic flavin semiquinone redox state was proposed as the active signaling conformation [15, 16].

Controversy involving this mechanism revolved around the observation that mutants involved in electron transfer to the flavin in DmCry which fail to photoreduce *in vitro* nonetheless retained biologically activity *in vivo* [13, 17]. This was taken as evidence that formation of the anionic radical (FAD^•-^) redox state is not required for biological signaling. However, this apparent contradiction is resolved by the demonstration that mutants which fail to undergo photoreduction *in vitro* are nonetheless photoreduced *in vivo* [16, 18, 19], indicating that formation of the anionic radical redox state occurs by alternate electron transfer routes and therefore can initiate signaling. Further controversy has arisen on apparent methodological grounds [compare e.g. 15, 20], and on whether redox changes could explain the light sensitivity of DmCry responses and half-life of the signaling states *in vitro* [13, 17]. This has prompted the suggestion of a mechanism of DmCry activation where the anionic radical (FAD^•-^) instead of oxidized FAD, may be the light-absorbing species, which undergoes some unspecified photoreaction [17, 20].

Recently, it has been shown that plant cryptochromes produce reactive oxygen species (ROS) as a result of illumination [21,22]. This follows from observations *in vitro* that a consequence of the *Arabidopsis* cryptochrome flavin redox cycle is the formation of ROS and hydrogen peroxide (H_2_O_2_) in isolated proteins [23,24]. Specifically, *Arabidopsis* cry1 and cry2 both undergo flavin reduction from FAD_ox_ to a mixture of radical (FADH^•^) and reduced (FADH^-^) redox states as a consequence of illumination. Upon return to darkness, these proteins are reoxidized to the FAD_ox_ redox state by a process that releases superoxide and hydrogen peroxide both *in vitro* [23] and *in vivo* [21,22]. ROS accumulation occurs in the nucleus where cryptochromes are localized, in contrast to mitochondrial and cytoplasmic compartments which produce ROS via metabolic enzymes.

To help resolve existing controversies concerning DmCry activation and also to provide novel insights into the oxidative signaling mechanism of DmCry, we have here re-examined the DmCry photocycle of flavin reduction/reoxidation in detail by a kinetic modeling approach. This allowed us to calculate the quantum efficiency of photon absorption and show that this reaction has the light sensitivity to serve a signaling role. We further determined the half-life of the presumed signaling state (FAD^•-^) *in vitro* and show that it is consistent with published estimates of the lifetime of the signaling state of DmCry *in vivo* [17, 20]. Finally, we have added a new dimension to the DmCry signaling paradigm by demonstrating the formation and accumulation of intracellular ROS as a result of illumination. These results suggest the possibility that direct enzymatic production of ROS by cryptochrome may represent an alternate oxidative signaling mechanism that may be conserved across phylogenetic lines.

## Materials and methods

### Protein samples and photoreduction experiments

**Fig 1:**
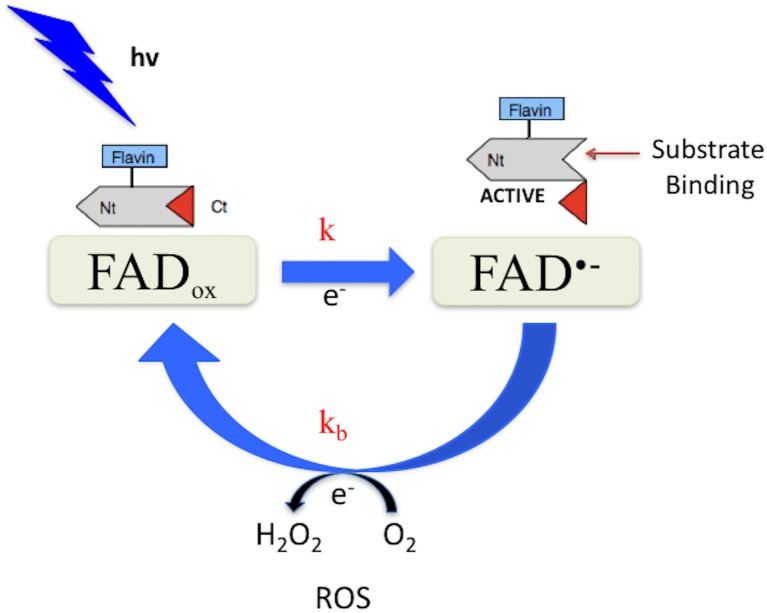
Possible *Drosophila* cryptochrome photocycle. In the dark, the protein-bound cofactor (FAD_ox_) is shown in the oxidized redox state. Light absorption triggers flavin photoreduction [12 – 14] at a rate constant k. Reoxidation to the FAD_ox_ state occurs spontaneously in the dark at a rate constant k_b_. Possible changes in C-terminal conformation linked to redox state interconversion are diagrammed [15].

**Fig 2:**
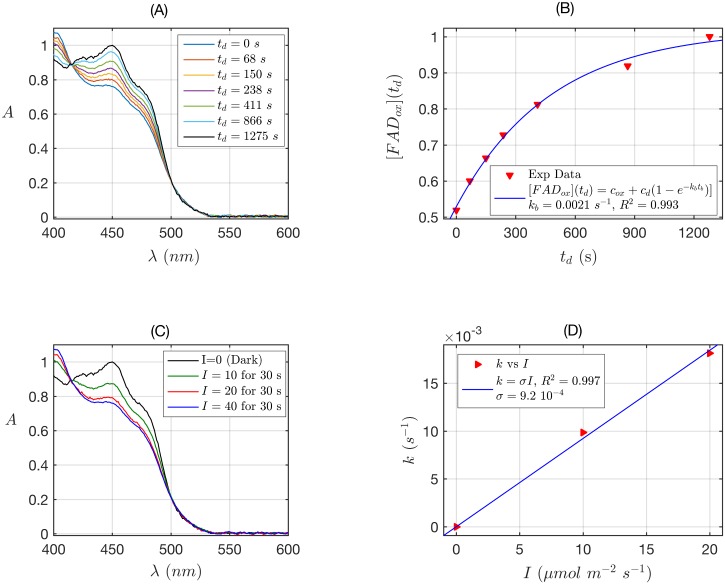
Rate constants and quantum yield for two-state reduction and reoxidation of DmCry. (A) Isolated purified DmCry protein was illuminated for 30 s at I = 40 μmol m^-2^ s^-1^ blue light and placed in darkness (t_d_ = 0 s). Normalized absorption spectra are reported at increasing dark reoxidation times t_d_. (B) Normalized concentration of FAD_ox_ as a function of the dark reoxidation time t_d_. The FAD_ox_ concentration was obtained from the absorbance at 450nm from panel A according to Eq. S7 (see S1 Text). The red triangles represent the experimental data, and the blue curve is the fit of the experimental data with the two-states reoxidation model (Eq. S8). From the fit the reoxidation rate resulted k_b_ = 0.0021 s^-1^, (half-life of τ_1/2_ = 5.5 min). The goodness of the fit was excellent (*R*^2^ ≈ 1). (C) Isolated purified DmCry protein was illuminated for 30 s at the indicated blue light fluence rates I. Normalized absorption spectra are presented. (D) Calculated forward rate constant k versus photon fluence rate I (red triangles). For each I, the rate constant k was calculated by numerically solving the two-states kinetic equations (see S1 Text, Eq. S1), with the concentration of FAD_ox_, obtained from panel C and reoxidation k_b_ obtained from panel B. We fit k as function of I by using the linear equation k = σ I. The fit is reported as blue curve in Fig 2. From the fit the photo-conversion cross section was σ = 9.2 x 10^−4^ μmol^-1^ m^2^. From σ the quantum yield ϕ was calculated according to σ = 2.3 ε_ox_(450) ϕ, by using the experimentally calculated extinction coefficient ε_ox_(450) = 1130 mol^-1^ m^2^ (11300 M^-1^ cm^-1^). The quantum yield resulted ϕ = 0.35.

**Fig 3:**
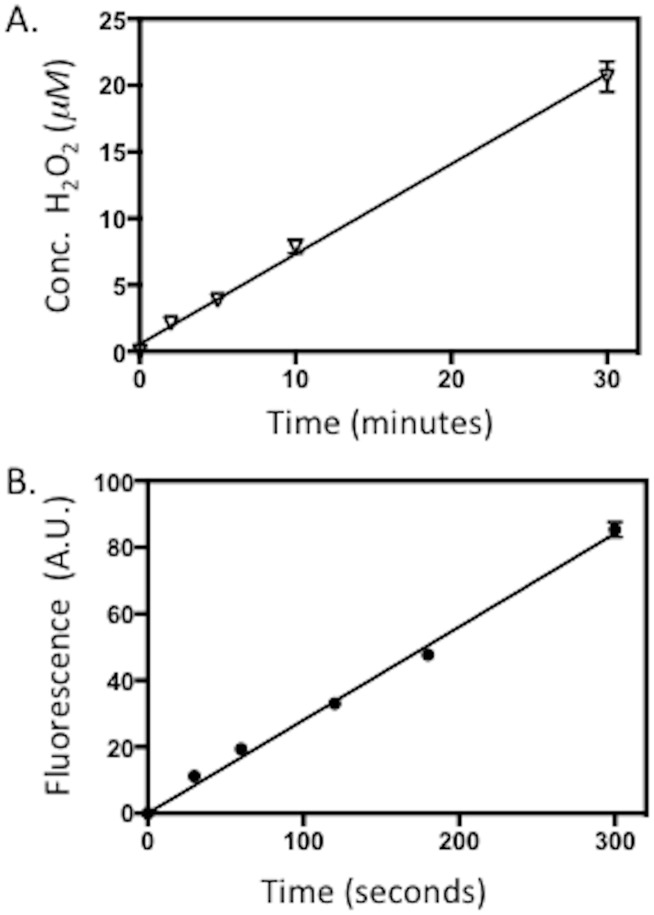
Formation of ROS by purified *Drosophila* Cryptochrome (DmCry). 30 μ*M* DmCry protein was illuminated at saturating blue light intensity for the indicated times on ice. A. The concentration of H_2_O_2_ released in the sample after illumination was determined by the Amplex Red fluorescence detection (see Methods). B. ROS formation in the course of illumination assayed by DCFH-DA fluorescence (ex: 490/em:530) (see Methods). Error is SD of three measurements.

These methods were used for results presented in Figs 1, 2 and 3. The full-length *Drosophila* cryptochrome cDNA sequence was cloned into the pAcHLT-A baculovirus transfer vector (BD Biosciences, San Jose, Ca.) in-frame with the N-terminal 6-His tag. The DmCry protein was expressed in Sf21 insect cell cultures by established methods and purified over an NTA nickel affinity column as previously described [25]. Photoreduction experiments were performed at 21°C in a buffer of 50mM phosphate, pH 7.5 and 10*mM* β-mercaptoethanol. A control expression construct (Spa1) consisted of the full-length *SPA1* cDNA [26] cloned into the pDEST10 baculovirus transfer vector (Thermo Fisher Scientific, Waltham, Ma.) and introduced for expression in Sf21 insect cells. Spa1 was chosen as it is involved in light signaling in plants but has no photoactive pigment and is not directly responsive to light [27].

### Kinetic analysis

Kinetic analysis and numerical methods for determination of quantum efficiency and half-life were performed as described previously [28] using optical spectra from isolated DmCry. Details of the present analysis are included in the Supplementary Material (S1 Text).

### Detection of ROS in purified protein samples

For determination of ROS, DmCry protein at a concentration of 30 *μM* in PBS at pH 7.4 and in the absence of added reducing agent was illuminated with blue light (3000 μmol m^-2^ sec^-1^) for 30 minutes at 0°C. Aliquots were taken at the indicated times and frozen into liquid nitrogen prior to ROS determination. *H*_*2*_*O*_*2*_ *detection*: 3*μ*l of protein sample was diluted into 0.3 ml of 50 mM of sodium phosphate buffer at pH 7.4 and adjusted to a final concentration of 10 *μM* Amplex UltraRED (Invitrogen/Thermo Fisher Scientific, Waltham, Ma.) and 0.2 U of horse radish peroxidase (Sigma Aldrich, St Louis, Mo. USA). After 30 minutes of incubation time in the dark, fluorescence was read in triplicate from each sample (100 μl volume for each reading) in 96 well plates with a Cary Eclipse fluorescence spectrophotometer (Varian) at absorption 560 nm, emission 590 nm. Fluorescence units were converted to concentration of H_2_O_2_ by a standard curve of concentration vs. fluorescence units as described previously [21]. The H_2_O_2_ concentration displayed on the Y-axis of the graphical representation refers to the total concentration of H_2_O_2_ in the undiluted protein sample. *ROS detection using dichlorofluorescein fluorescent substrate*: Transient formation of ROS was monitored by addition of 1 *mM* CM-H_2_DCFDA (5,6-chloromethyl-2,7-dichlorodihydrofluorescein diacetate, Molecular Probes, Life Technologies, Grand Island, NY, USA) to the protein samples immediately prior to illumination. In the text, we have abbreviated the name of this reagent to DCFH-DA. The fluorescence was read in triplicate from each sample (100 μl undiluted sample volume for each reading) in 96 well plates with a Cary Eclipse fluorescence spectrophotometer (Varian) at excitation/emission of 490/530nm.

### Detection of ROS in Sf21 insect cell culture

**Fig 4:**
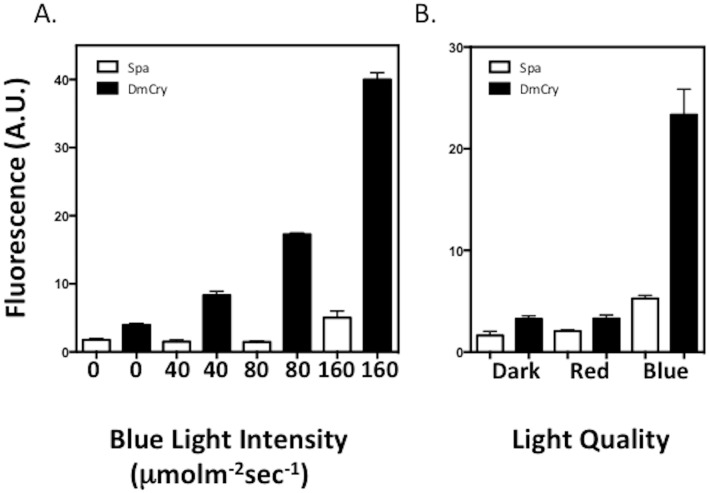
Induction of ROS in insect cell culture expressing DmCry. A. Living insect cell cultures expressing either DmCry or a SPA1 control construct were treated with the fluorescent substrate DCFH-DA and then exposed to the indicated blue light intensity for 10 min. Subsequently to illumination, DmCry expressing and control cell cultures were harvested, lysed, and evaluated by fluorescence spectroscopy for the formation of ROS (see Methods). B. Cells exposed to dark, red, or blue light. Error bars represent SD of three measurements.

Insect cells expressing either DmCry or Spa1 expression constructs were harvested 72 hours post-infection and resuspended in PBS buffer (50 mM sodium phosphate, 150 mM NaCl buffer pH 7.4) at a final concentration of 2 x 10^5^ cells/ml. DCFH-DA was then added to a final concentration of 1 *mM* prior to illumination at 22°C for the indicated times and light qualities (Fig 4). Illumination was in 24-well microtitre plates placed directly under the light source for 10 minutes, with 1 ml cell cultures per well. Subsequent to illumination, cells were harvested, washed twice in PBS, then lysed in a final volume of 0.5m l PBS with the addition of 0.1% Triton X-100. 80 *μ*l aliquots of the whole cell lysate were transferred to individual wells of a 96 well microtitre plates and measured at excitation/emission of 490/530nm.

### Immunofluorescence labelling of DmCry

Sf21 cells incubated during 2 hours on glass coverslips were exposed to dark or blue light for 15 min and fixed with 2% paraformaldehyde for 10 min at room temperature (RT), permeabilized with 0.1% Triton X-100 and then incubated with an anti-DmCry rabbit polyclonal antibody [28] [16] and an Alexa 488-conjugated anti-rabbit secondary antibody. Coverslips were mounted in Fluoroshield with DAPI (4’, 6’-diamino-2-phenylindole) and viewed by a Leica upright SP5 confocal microscope with a 63X objective. DAPI and Alexa 488 were, respectively, excited at a 405 and 488 nm wavelengths, and the Emission fluorescence intensities and DIC were detected by using a photomultiplicator between 498 and 561 nm, and a transmission photomultiplicator, respectively. Two channels were recorded sequentially at each z-step. Z series projections and merge images were performed using ImageJ software (W. S. Rasband, ImageJ [U.S. National Institutes of Health, Bethesda, MD; http://rsb.info.nih.gov/ij, 1997–2009]).

### Intracellular localization of ROS

Sf21 living cells expressing DmCry or the control *SPA1* construct were washed 2 times in PBS (pH 7.4) and incubated in PBS containing 58 μM DCFH-DA (Molecular Probes, Life Technologies, Grand Island, NY, USA) for 10 min in the dark, then exposed to blue light during 5 min and observed immediately either between glass and coverslips with a Zeiss AxioImager.Z1/ApoTome microscope or in an observation chamber with an inverted Leica TCS SP5 microscope. Green fluorescence from DCFH-DA was excited at 488 nm. Zeiss AxioImager.Z1/ApoTome observations were done by using a 10X objective. Emission fluorescence intensities were detected by using the Zeiss filter set 38 Endow GFP shift free; EX BP 470/40, BS FT 495, EM BP 525/50 and differential interference contrast (DIC) with an Analy DIC TransLight. The images were digitally captured with a CCD-camera (AxioCam MRm) using the software Axiovision (version 4.7.2, Carl Zeiss).

Inverted Leica TCS SP5 microscope observations were done by using a 40x objective. Emission fluorescence intensities were detected by using a photomultiplicator between 498 and 561 nm and DIC by using a transmission photomultiplicator. Z series projections were performed using ImageJ software (W. S. Rasband, ImageJ).

## Results

### Kinetic modeling of the *Drosophila* cryptochrome photocycle

Purified preparations of DmCry protein have been shown to undergo a photoreduction reaction *in vitro* [12 – 14]. This involves transition from the FAD cofactor bound in the oxidized redox state (FAD_ox_) to the anionic radical (FAD^•-^). Upon return to darkness, the flavin spontaneously re-oxidizes in the presence of molecular oxygen to restore the resting (FAD_ox_) state, giving rise to a continuous photocycle under constant illumination (Fig 1). Therefore, the concentration of the FAD^•-^ flavin radical redox state depends on the rate constants k and k_b_ according to the two-states kinetic model (see S1 Text in Supplementary Material). **T**his light-dependent redox reaction has been linked to biological activity in a number of studies [15, 16]. However, the kinetic parameters of the reaction (light sensitivity, lifetime of redox state intermediates, and quantum yield) have yet to be rigorously established.

We therefore first derived a two-states kinetic model of the DmCry photocycle. Photoreduction experiments were performed by illuminating purified DmCry protein samples at increasing intensities (photon fluences I) of blue light for 30 seconds in the presence of a mild reductant (10*mM* β-mercaptoethanol) (Fig 2). Flavin reduction to the anionic radical (FAD^•-^) redox form could then be observed by spectral decrease at 450nm.

We have estimated the dark reoxidation rate k_b_ (dark reversion time) for reoxidation of flavin from the FAD^•-^ back to FAD_ox_. For this experiment, DmCry protein samples were illuminated at maximum light intensity and then returned to darkness. Spectra were taken at defined dark intervals t_d_ (Fig 2A). From these data it was possible to estimate the reoxidation half-life (see [28] and S1 Text). Fig 2B reports (red triangles) the normalized FAD_ox_ concentration calculated from panel (A) as a function of the dark time t_d_, and (blue curve) the fit of the data with the two-states dark reoxidation model equation reported in the legend (Eq. S7). The dark reoxidation rate resulted k_b_ = 0.0021 s^-1^, which gives a half-life of τ_1/2_ = 5.5 minutes.

Calculation of the quantum yield for flavin reduction was performed using the data obtained from spectra reported in Fig 2A and 2B. A two-state kinetic model was used as described previously for Arabidopsis cry [28]—see also detailed description of the methods in S1 Text. Fig 2D shows (red triangles) the forward rate constant k as a function of the blue light intensity (fluence rate) I used in panel (C), and (blue curve) the linear fit k = σ I of the data. From the linear fit we estimated a photoconversion cross section of σ = 9.2 10^−4^ (in μmol^-1^ m^2^ units), which allowed us to calculate a quantum yield of ϕ = 0.35 by using an estimated molar extinction coefficient of ε_ox_(450) = 11300 M^-1^ cm^-1^ [29] (for details of calculations see [28] and S1 Text).

The quantum yield for flavin reduction of 0.35 is well within the range for biological signaling molecules and comparable with that reported for *Arabidopsis* cry2 of 0.19 [28]. The half-life of the FAD^•-^ redox state is also comparable to that of *Arabidopsis* cry1 and cry2, where photoreduction leads to formation of the neutral radical (FADH^•^) redox state [28]. DmCry by contrast forms the charged anionic radical (FAD^•-^) both *in vitro* or *in vivo* [14, 16]. These data suggest a significant stabilizing effect of the intraprotein environment on the flavin radical in DmCry.

### DmCry illumination induces the formation of ROS

One of the characteristics of flavin reoxidation from the radical (FADH^•^) or reduced (FADH^-^) to the oxidized (FAD_ox_) redox state in plant cryptochromes is the transient formation of ROS, producing superoxide and hydrogen peroxide (H_2_O_2_) [21 – 23]. To determine whether DmCry activation likewise induces the formation of ROS, we have illuminated isolated samples of DmCry and tested for the production of H_2_O_2_ using a resorufin fluorometric assay.(see Methods). The resorufin fluorescence detection is a selective measurement of H_2_O_2_, which is a secondary byproduct of possible superoxide (O_2_^•-^) or other radical formation [23] Aliquots of DmCry were removed at the indicated times and the concentration of H_2_O_2_ determined (Fig 3A). The concentration of H_2_O_2_ increased in a linear fashion over a time period of 30 min (Fig 3A).

To directly assay for short-lived ROS such as O_2_^•-^ or other intermediate ROS, we further analysed the protein samples with a general indicator of ROS formation, the fluorescent probe DCFH-DA [30]. In this assay, the fluorescence substrate DCFH-DA was added to the protein immediately prior to illumination (Fig 3B). Aliquots were analysed at the given time points for fluorescence resulting from the formation of ROS. Illumination indeed caused a linear increase over time in signal for DmCry (Fig 3B), whereas control protein samples at the same concentration such as BSA showed no increase (not shown). We conclude the signal is due largely to the production of ROS as a result of DmCry flavin reoxidation.

### DmCry illumination induces the formation of ROS in living cells

To determine that DmCry illumination also leads to the induction of ROS in living cells we analysed Sf21 insect cell cultures expressing recombinant DmCry from baculovirus expression constructs. For this assay, the fluorescent substrate DCFH-DA was added to the cell incubation medium prior to illumination (see Methods). As a negative control, we used Sf21 insect cells expressing a different construct lacking photoactive pigments, namely *SPA1* (Suppressor of Phy A), a plant protein that is implicated in light responsivity in plants but is not photochemically active [26, 27]. This control was used to correct for possible non-specific effects on ROS induction, since viral infection and recombinant protein expression could of themselves initiate stress response that is unrelated to DmCry activation.

The results showed that illumination of DmCry expressing cell cultures induced a clear increase in ROS production compared to control cell cultures (Fig 4A). This effect was observable already as low as 40μmol m^-2^ sec^-1^ blue light, and increased with increasing illumination (Fig 4A). At very high light intensities (160 μmol m^-2^ sec^-1^) ROS formation also increased modestly in the negative control cell (*SPA1* expressing) cultures, indicating non-specific effects of blue light illumination on cellular stress. Induction of ROS was not observed in darkness or red light (Fig 4b), consistent with a requirement for activation of cryptochrome.

### DmCry illumination induces formation of ROS in the nucleus

**Fig 5:**
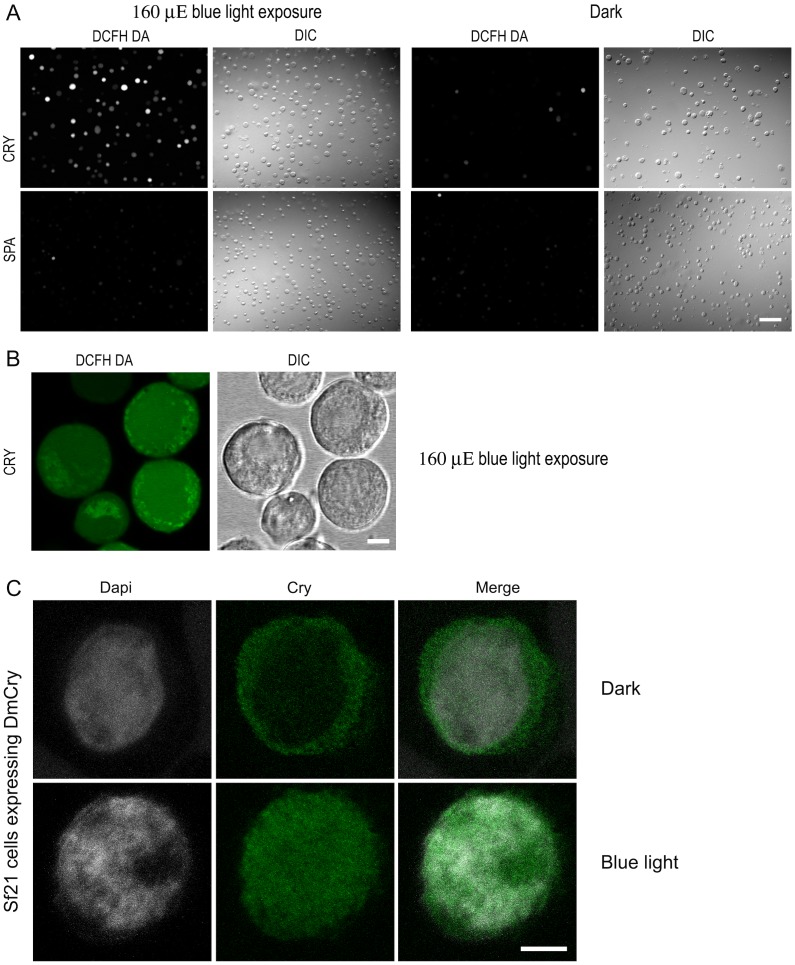
Subcellular localization of ROS and DmCRY in Sf21 insect cells exposed to blue light. Living Sf21 cells stably expressing DmCRY were treated with DCFH-DA, exposed to dark or blue light and viewed by **(A)** a Zeiss AxioImager.Z1/ApoTome using a 10x objective (bar 100 μm) **(B)** an inverted Leica TCS SP5 microscope. Images show single confocal z section that cross the nucleus. Diffused fluorescent ROS staining can be seen in nucleus and cytoplasm. Punctuate and intense fluorescent ROS staining also colocalizes perfectly with ER (endoplasmic reticulum) surrounding the nucleus. Scale bar: 10 μm. **(C)** Sf21 stably expressing DmCry were fixed with paraformaldehyde, permeabilized with Triton X100, incubated with an anti—DmCry1 rabbit polyclonal antibody and an Alexa 488—conjugated anti—rabbit secondary antibody, DNA were stained with 4′,6′ — diamino—2—phenylindole (DAPI). Cells were observed with a Leica TCS SP5 confocal microscope. Images show projections of optical sections that cross the nucleus. Scale bar, 10 μm.

Information on the localization of ROS was obtained through staining of DmCry expressing cell cultures with DCFH-DA during illumination (see Methods). After 10 minutes blue light illumination, DmCry expressing cells showed a significant increase in fluorescence as compared to control cells (expressing the SPA1 construct) in blue light, but not in darkness (Fig 5A). This validates our biochemical data (Fig 4) and confirms that rapid ROS induction is a consequence of DmCry illumination *in vivo*. To obtain details of intracellular localization, confocal microscopy was used after blue light exposure (Fig 5B). Diffuse fluorescence could be seen throughout the cell but was also localized within the nuclear compartment. Particularly pronounced vesicular structures are likely endoplasmic reticulum surrounding the nucleus, and may arise as a consequence of the cell’s attempts to remove excess ROS by secretion into the extracellular medium.

To obtain information concerning the localization of DmCry, we performed immunostaining with anti-DmCry antibody. In dark-adapted cells, staining can be seen primarily in the cytosol and is largely absent in the nucleus (Fig 5C), consistent with DmCry localization in the cytosol. After 15min illumination with 80 μmoles m^-2^sec^-1^ blue light, immunostaining of these same cell cultures showed significant increase in cryptochrome localization in the nucleus. These data indicate that DmCry is localized primarily in the cytoplasmic compartment in the dark, but moves into the nuclear compartment upon activation. Similar results were previously obtained in the case of the *Arabidopsis* cry2 [22].

In sum, these data show colocalization of ROS biosynthesis with DmCry protein expression, and are thereby consistent with primary synthesis of ROS by cryptochromes in living cells.

## Discussion

One of the key questions in DmCry activation concerns the nature of the photoreactions that are implicated in biological activity. Numerous studies have linked a redox state interconversion to signaling in DmCry, including by action spectroscopy that shows oxidized flavin as the photosensor in the dark-adapted state [16,31], that flavin photoreduction accompanies light activation [12,14], and that redox change of the flavin *in vitro* can induce conformational change and productive interaction with substrate proteins even in the absence of light [15].

However, there has also been a great deal of controversy concerning this mechanism. Much of the confusion has resulted from the observation that mutants of DmCry that do not undergo flavin reduction *in vitro* [13, 17, 20] have been reported to retain biological activity *in vivo*, thereby disputing that formation of the flavin radical is required for biological function. This observation can be explained by the fact that cryptochrome mutants defective in electron transfer *in vitro* are nevertheless photoreduced *in vivo* by alternate routes [16,18,19]. Therefore they indeed form the flavin radical redox state in response to light *in vivo* and their observed biological activity is expected. Confusion concerning the role of flavin reduction in has been further exacerbated by studies that incorrectly analysed cryptochrome mutant phenotypes, either by scoring constitutive dark phenotypes as ‘light activated’ or performing experiments far above saturating light intensities such that differential responsivity was missed (for full discussion of recent literature, see [15]). Nonetheless, a signaling mechanism based on activation by redox state interconversion has not been conclusively demonstrated and one of the weaknesses has been that the DmCry photocycle has not been rigorously characterized and correlated to DmCry signaling events.

For this reason, one of the main goals of the present study was to ascertain whether a photocycle based on flavin reduction (Fig 1) is indeed compatible with known characteristics of *in vivo* signaling by DmCry. It should be cautioned in this context that there can be considerable variation in reported light sensitivity of DmCry dependent phenotypes *in vivo*, which is not necessarily linked to the actual light sensitivity of the receptor. For instance, light-induced proteolysis of DmCry and signaling in neuronal firing [8, 17] require much higher apparent irradiance than phase shifting of the circadian clock [32]. Such variability is a classic feature of biological signaling reactions, which result from signal amplification events that are far downstream of the receptor [33]. Nonetheless, the quantum yield obtained for flavin reduction of 0.35 is well within the range for biological signaling molecules and is comparable with the quantum efficiency of both *Arabidopsis* cry2 [28] and phot1 [34], which are both sensitive plant flavoprotein receptors operating in a low fluence range. The fact that flavin reduction is efficient and occurs in response to relatively low light intensity (Fig 2) is consistent with a signaling role *in vivo*.

Another kinetic feature of the DmCry photocycle consistent with a biological signaling role is the relatively long half-life (5.5 min) of the anionic radical (FAD^•-^) redox state. This indicates that, if the anionic radical redox state is indeed the activated signaling state of DmCry, then biological activity is predicted to persist for several minutes after the end of illumination. Indeed, proteolytic experiments have shown that even a single flash of light of less than 1 msec is sufficient to induce biological activation of DmCry, which however is only apparent after a delay of several minutes in the ensuing dark interval [13]. Other studies suggest a half-life of up to 15 minutes for the signaling state of DmCry *in vivo*, as estimated by the lifetime of the activated conformational state [17], consistent with the extended lifetime of the anionic radical (FAD^•-^) redox state shown in this study.

The second finding presented here is the demonstration that ROS are formed upon photoactivation of DmCry. A possible mechanism for the one electron reduction of O_2_ by FAD^•-^ and subsequent production of H_2_O_2_ is shown in the reaction scheme (S1 Fig) included in the Supporting Information. The dismutation of superoxide would be facilitated at pH7.4 by the presence of small amounts of hydroperoxy radical, leading to H_2_O_2_ and O_2_ as final products.

In this reaction scheme (S1 Fig), every time the flavin in DmCry becomes reduced by light, there is a molecule of H_2_O_2_ formed during the subsequent reoxidation step. The rate of this reoxidation (*k*_b_–Fig 1 of main text) is not dependent on light and so occurs at a constant rate during illumination. Thus, illumination of DmCry should result in synthesis of ROS in proportion to the concentration of the protein, the extent of flavin reduction (dependent on the light intensity), and the overall illumination time. In keeping with this expectation, the concentration of H_2_O_2_ formed by DmCry *in vitro* increased linearly over time and was indeed proportional to the protein concentration (see Fig 3a, which reports the concentration of H_2_O_2_ formed over a 30 minute time period by a protein sample at 30 μmolar concentration). Furthermore, the DCFH-DA fluorescent substrate, which detects O_2_^•-^ in addition to other ROS products, likewise showed a linear increase in ROS subsequent to illumination of the isolated protein (Fig 3B).

Significantly, light-induced formation of ROS could also be detected in living cells, and co-localized with DmCry in both cellular and nuclear compartments of Sf21 insect cells (Fig 5). Since ROS production is a direct enzymatic property of DmCry illumination irrespective of cellular partner proteins and cofactors (see Fig 3), it should occur even at the lower DmCry protein concentration in the natural cellular evnvironment. These results, taken together with prior in-cell EPR spectroscopy [16], show that cycles of flavin reduction/reoxidation must occur *in vivo* in response to continuous DmCry illumination under physiological conditions. DmCry flavin must furthermore be in the oxidized (FAD_ox_) redox state in the dark for this to occur, which contradicts an alternate suggestion that the anionic radical redox state may represent the resting, dark-adapted state of DmCry [13, 20] and undergoes some unspecified photoreaction.

Finally, our results present the intriguing possibility that enzymatic biosynthesis of ROS by DmCry may contribute to its signaling role. It should be emphasized that the conformational change in DmCry triggered by flavin reduction occurs well before the reverse (reoxidation) reaction that generates ROS, and therefore it may be difficult to determine which signaling effects are due solely to ROS formation. Nonetheless, ROS in and of itself is an important regulator of cellular stress and ageing across phylogenetic lines [35]. A recent report in *Drosophila* indicates that restoring normal levels of CRY in ageing flies restores normal rhythmicity and improves longevity [36], whereas novel DmCry responses have been linked to regulation of genes implicated in stress response and ROS signaling [37]. It is not excluded that some of these effects may be due to activation of redox sensitive transcription factors by ROS synthesized by DmCry Alternatively, a recent intriguing report has shown modulation of redox activated potassium channels of the plasma membrane by drosophila cryptochromes. This suggests a possible ROS signaling role of DmCry that may involve ROS-dependent activation of a cytosolic redox sensitive substrate [38].

## Supporting information

S1 Text Supplement to methods for kinetic modelling of DmCry photocycle. (DOCX)

S1 Fig A possible mechanism for the one electron reduction of O_2_ by FAD^•-^ and subsequent production of H_2_O_2_. (TIFF)
