# Supplementary material for: Blue-light induced accumulation of reactive oxygen species is a consequence of the Drosophila cryptochrome photocycle
Source: PLoS One. 2017 Mar 15;12(3):e0171836. doi: 10.1371/journal.pone.0171836 (PMC5351967; doi:10.1371/journal.pone.0171836)
Supplement: S1 Text — (DOCX) [file pone.0171836.s001.docx]

Supplementary Material

**Blue-light induced accumulation of Reactive Oxygen Species is a consequence of the *Drosophila* cryptochrome photocycle**.

Louis-David Arthaut^1,2^, Nathalie Jourdan^1^, Ali Mteyrek^3^, Maria Procopio^1,4^, Mohamed El-Esawi ^1,5^, Alain d’Harlingue^1^, Pierre-Etienne Bouchet^1^, Thorsten Ritz^4^, André Klarsfeld^3^, Serge Birman^3^, Robert J. Usselman^6^, Ute Hoecker^7^, Carlos F. Martino^2^ and Margaret Ahmad^1,8^.

Short title: Cryptochrome synthesis of ROS.

^1^ UMR CNRS 8256 (B2A), IBPS, Université Paris VI, Paris, 75005 France

^2^Department of Biomedical Engineering, Florida Institute of Technology, Melbourne, FL 32901 U.S.A.

^3^GCRN team, Brain Plasticity Unit, UMR 8249 CNRS/ESPCI Paris, PSL Research University, 75005 Paris, France..

^4^Department of Physics and Astronomy, University of California, Irvine, CA, 92697-4575 U.S.A.

^5^Botany Department, Faculty of Science, Tanta University, 31527 Tanta, Egypt.

^4^ Department of Physics and Astronomy, University of California, Irvine, CA, 92697-4575 U.S.A.

^5^ Botany Department, Faculty of Science, Tanta University, 31527 Tanta, Egypt.

^6^ Department of Chemistry and Biochemistry, Montana State University, Bozeman MT 59717 U.S.A.

^7^ Botanical Institute and Cluster of Excellence on Plant Sciences (CEPLAS), Biocenter, University of Cologne, 50674, Cologne, Germany.

^8^ Department of Biology, Xavier University, Cincinnati, Ohio, 45207, U.S.A.

* Corresponding author

E-mail: [margaret.ahmad@upmc.fr](mailto:margaret.ahmad@upmc.fr) (MA)

SI Text. Supplement to methods for kinetic modelling of DmCry photocycle.

# Kinetic model of DmCry

Drosophila cryptochromes (DmCrys) undergo light-induced flavin reduction from oxidized (${FAD}_{ox}$) to radical (${FAD}^{\bullet-}$) redox states, with subsequent re-oxidation upon return to darkness. Figure 1 reports the DmCry photocycle. The time evolution of the two intermediate state concentrations is described by a set of coupled first-order kinetic equations (Espensen 1981):

$$\left\{ \begin{aligned} \frac{d[{FAD}_{ox}]}{dt}=-k\left[ {FAD}_{ox} \right]+k_{b}[{FAD}^{\bullet-}] \\ \frac{d[{FAD}^{\bullet-}]}{dt}=k\left[ {FAD}_{ox} \right]-k_{b}[{FAD}^{\bullet-}] \end{aligned} \right. (S1)$$

where k is the forward rate constant, and k_b_ the dark re-oxidation rate constant. In Eq. S1 square brackets denote the normalized concentrations of the transient states ${FAD}_{ox}$ and ${FAD}^{\bullet-}$. At any given time t, $\left[ {FAD}_{ox} \right]\left( t \right)+{[FAD}^{\bullet-}]\left( t \right)=1$, and the initial condition at t=0 is $\left[ {FAD}_{ox} \right]\left( 0 \right)=1$.

The analytical solution of the two-state kinetic model, reported in Eq. S1, is straightforward and results:

$$\begin{matrix} \left[ {FAD}_{ox} \right]\left( t \right)=\frac{k_{b}}{k+k_{b}}+\frac{k}{k+k_{b}}e^{-\left( k+k_{b} \right)t} \\ \end{matrix} (S2)$$

$$\begin{matrix} \left[ {FAD}^{\bullet-} \right]\left( t \right)=\frac{k}{k+k_{b}}({1-e}^{-\left( k+k_{b} \right)t} ) \\ \end{matrix} (S3)$$

We follow the method used in (Procopio, et al. 2016) to fit the two-state kinetic model to absorption spectra of the isolated proteins. This will allow to find the re-oxidation rate constants and quantum yields and thus predict the concentrations of the two states at a given illumination condition.

We record absorption spectra from 400 to 600 nm, by illuminating DmCry samples with blue light (450 +/-10nm). We apply the Beer-Lambert law, which relates the concentration of the transient states to the absorbance *A* (Schmidt, 2005). According to the Beer-Lambert law, the absorbance *A* at a given wavelength λ and at time *t* is linearly dependent on the concentration of the absorbing species (Schmidt 2005). In the frequency range considered here, both ${FAD}_{ox}$and ${FAD}^{\bullet-}$absorb blue light (450nm) (Bouly, et al. 2007, Banerjee, et al. 2007, Liu, Zhong and Lin 2010).

Thus there are two absorbing species at 450nm, and the Beer-Lambert results:

$A\left( 450,t \right)=\varepsilon_{ox}\left( 450 \right)\left[ {FAD}_{ox} \right]\left( t \right)+\varepsilon_{d}\left( 450 \right)\left[ {FAD}^{\bullet-} \right]\left( t \right)$ (S4)

where $\varepsilon_{ox}\left( 450 \right)$ and $\varepsilon_{d}\left( 450 \right)$ are the extinction coefficients of respectively ${FAD}_{ox}$ and

${FAD}^{\bullet-}$. The extinction coefficient of the anionic radical ${FAD}^{\bullet-}$ is about half the extinction coefficient of ${FAD}_{ox}$ $CITATION Liu10 \backslash l 1033 (Liu, Zhong and Lin 2010)$:

$$\varepsilon_{d}\left( 450 \right)=\frac{\varepsilon_{ox}\left( 450 \right)}{2} (S5)$$

We substitute Eq. S5 in Eq. S4, and then normalize the new expression to the dark, i.e.$A\left( 450,0 \right)=\varepsilon_{ox}\left( 450 \right)\left[ {FAD}_{ox} \right]\left( 0 \right)$. We re-define A(450,t) as the normalized absorbance and, ${[FAD}_{ox}]\left( t \right)$and $\left[ {FAD}^{\bullet-} \right]\left( t \right)$as the normalized concentration. Thus we obtain a simplified relationship between absorbance and concentration of the two species:

$$A\left( 450,t \right)=\left[ {FAD}_{ox} \right]\left( t \right)+\frac{\left[ {FAD}^{\bullet-} \right]\left( t \right)}{2} (S6)$$

Considering that at a given time t $\left[ {FAD}_{ox} \right]\left( t \right)+\left[ {FAD}^{\bullet-} \right]\left( t \right)=1$ we obtain from Eq. S6 the concentration of $\left[ {FAD}_{ox} \right]\left( t \right)$ and $\left[ {FAD}^{\bullet-} \right]\left( t \right)$ as a function of the absorption at 450 nm at a given time t as:

$\left[ {FAD}_{ox} \right]\left( t \right)=2A\left( 450,t \right)-1 (S7)$

$$\left[ {FAD}^{\bullet-} \right]\left( t \right)=2-2A\left( 450,t \right) (S8)$$

We use Expressions S7 and S8 to fit absorption spectra with the kinetic model, and find re-oxidation rate k_b_ and quantum yield φ (Procopio, et al. 2016).

## Dark re-oxidation rate k_b_

### Since flavin re-oxidation from $\boldsymbol{FAD}^{\boldsymbol{\bullet}\boldsymbol{-}}$to $\boldsymbol{FAD}_{\boldsymbol{ox}}$can be monitored spectroscopically by following the absorbance at 450nm, we first obtain experimental values for the dark re-oxidation kinetics from the anionic radical ($\boldsymbol{FAD}^{\boldsymbol{\bullet}\boldsymbol{-}}$) to the oxidized ($\boldsymbol{FAD}_{\boldsymbol{ox}}$) flavin states of the DmCry.

### We define the re-oxidation time as t_d_, and t_d_ = 0 is the time in which DmCry is placed in darkness, after being illuminated for a certain time t at fluence rate I. We record the absorption spectrum after increasing times in darkness t_d_, until complete re-oxidation to $\boldsymbol{FAD}_{\boldsymbol{ox}}$ (see Fig. 2A). When only dark re-oxidation occurs (k=0), the analytical solutions of Eq. S1 for $\left[ \boldsymbol{FAD}_{\boldsymbol{ox}} \right]\left( \mathbf{t} \right)$is:

$$\left[ {FAD}_{ox} \right]\left( t_{d} \right)=c_{ox}+c_{d}\left( 1-e^{-k_{b}t_{d}} \right) (S8)$$

${[{FAD}^{\bullet-}}]\left( t_{d} \right)=c_{d}e^{-k_{b}t_{d}}$ (S9)

where *c*_ox_ and *c*_d_ are the initial concentrations of, respectively, ${FAD}_{ox}$and ${FAD}^{\bullet-}$at the dark time t_d_=0. We plot the concentrations of ${FAD}_{ox}$, obtained from the spectra at 450nm by using Eq. S7, as a function of the dark recovery time t_d_, and fit the data with Eq. 3S (see Fig. 2B). We calculate the half-life **τ_1_**_/2_ according to τ_1/2_=ln (2) k_b_^-1^.

## Quantum yield φ

We calculate the quantum yield by exploiting the linear correlation between forward rate constant and fluence rate, i.e. k=σI, where σ is the photo-conversion cross section. The photo-conversion cross section σ is related to the quantum yield φ according to σ=2.3ε_ox_(450)φ (Kendrick and Kronenberg 1994). To obtain the photo-conversion cross section we illuminate DmCry sample with increasing blue light fluence rates I and record absorption spectra (see Fig. 2C). For each fluence rate I we calculate the concentration of ${FAD}_{ox}$from the spectra (Eq. S7), which we use to find the corresponding forward rate k by numerically solving Eq. 2 (see two-state model algorithm in (Procopio, et al. 2016)). We estimate σ by fitting I vs. k with a linear function, k=σI (see Fig. 2D). From σ we calculate φ by using the extinction coefficient ε_ox_(450) of DmCry (see main text).

# Bibliography

Banerjee, R., E. Schleicher, S. Meier, R.M. Viana, R. Pokorny, M. Ahmad, R. Bittl, and A. Batschauer. 2007. "The signaling state of Arabidopsis cryptochrome 2 contains flavin semiquinone." *J. Biol. Chem.* 282: 14916–14922.

Bouly, J.P., E. Schleicher, M. Dionisio-Sese, F. Vandenbussche, D. Van Der Straeten, N. Bakrim, S. Meier, et al. 2007. "Cryptochrome bluelight photoreceptors are activated through interconversion of flavinredox states." *J. Biol. Chem.* 282: 9383–9391.

Espensen, J. H. 1981. *Chemical Kinetics and Reaction Mechanisms.* New York: McGraw-Hill.

Kendrick, R.E., and G.H.M. Kronenberg. 1994. *Photomorphogenesis in Plants.* Springer Science & Business Media.

Liu, Hongtao, Dongping Zhong, and Chentao Lin. 2010. "Searching for a photocycle of the cryptochrome photoreceptors." *Curr Opin Plant Biol* 13 (5): 578–586.

Müller, P., and M. Ahmad. 2011. "Light-activated cryptochrome reacts with molecular oxygen to form a flavin-superoxide radical pair consistent with magnetoreception." *The Journal of Biological Chemistry* 286 (24): 21033-21040.

Procopio, M, J Link, D Engle, J Witczak, T Ritz, and M Ahmad. 2016. "Kinetic modeling of the Arabidopsis cryptochrome photocycle: FADHo accumulation correlates with biological activity." *Front. Plant Sci* 7: 888.

Schmidt, W. 2005. *Optical Spectroscopy in Chemistry and Life Sciences: An Introduction.* New York: Wiley-VCH.

# S1 Figure Captions

# SI Fig 1 Reaction scheme for flavin reoxidation. A possible mechanism for the one electron reduction of O_2_ by FAD^⦁-^ and subsequent production of H_2_O_2_ .
