# Supplementary figures and images for: Blue-light induced accumulation of reactive oxygen species is a consequence of the Drosophila cryptochrome photocycle
Source: PLoS One. 2017 Mar 15;12(3):e0171836. doi: 10.1371/journal.pone.0171836 (PMC5351967; doi:10.1371/journal.pone.0171836)

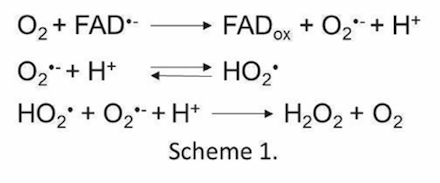

Supplement: S1 Fig — (TIFF) [file pone.0171836.s002.tiff]
